# A stitch in time: Combining more than two decades of mooring data from the central Oregon shelf

**DOI:** 10.1016/j.dib.2023.109041

**Published:** 2023-03-07

**Authors:** Craig M. Risien, Brandy T. Cervantes, Melanie R. Fewings, John A. Barth, P. Michael Kosro

**Affiliations:** College of Earth, Ocean, and Atmospheric Sciences, Oregon State University, Corvallis, OR 97331, USA

**Keywords:** Seawater temperature, Practical salinity, Water velocity, NH-10, Mooring, California current, Newport, Oregon

## Abstract

The highly biologically productive northern California Current, which includes the Oregon continental shelf, is an archetypal eastern boundary region with summertime upwelling driven by prevailing equatorward winds and wintertime downwelling driven by prevailing poleward winds. Between 1960 and 1990, monitoring programs and process studies conducted off the central Oregon coast advanced the understanding of many oceanographic processes, including coastal trapped waves, seasonal upwelling and downwelling in eastern boundary upwelling systems, and seasonal variability of coastal currents. Starting in 1997, the U.S. Global Ocean Ecosystems Dynamics – Long Term Observational Program (GLOBEC-LTOP) continued those monitoring and process study efforts by conducting routine CTD (Conductivity, Temperature, and Depth) and biological sampling survey cruises along the Newport Hydrographic Line (NHL; 44.652°N, 124.1 – 124.65°W), located west of Newport, Oregon. Additionally, GLOBEC-LTOP maintained a mooring slightly south of the NHL, nominally at 44.64°N, 124.30°W, on the 81-meter isobath. This location is referred to as NH-10, as it is located 10 nautical miles or 18.5 km west of Newport. A mooring was first deployed at NH-10 in August 1997. This subsurface mooring collected water column velocity data using an upward-looking acoustic Doppler current profiler. A second mooring with a surface expression was deployed at NH-10 starting in April 1999. This mooring included velocity, temperature and conductivity measurements throughout the water column as well as meteorological measurements. GLOBEC-LTOP and the Oregon State University (OSU) National Oceanographic Partnership Program (NOPP) provided funding for the NH-10 moorings from August 1997 to December 2004. Since June 2006, the NH-10 site has been occupied by a series of moorings operated and maintained by OSU with funding from the Oregon Coastal Ocean Observing System (OrCOOS), the Northwest Association of Networked Ocean Observing Systems (NANOOS), the Center for Coastal Margin Observation & Prediction (CMOP), and most recently the Ocean Observatories Initiative (OOI). While the objectives of these programs differed, each program contributed to long-term observing efforts with moorings routinely measuring meteorological and physical oceanographic variables. This article provides a brief description of each of the six programs, their associated moorings at NH-10, and our efforts to combine over twenty years of temperature, practical salinity, and velocity data into one coherent, hourly averaged, quality-controlled data set. Additionally, the data set includes best-fit seasonal cycles calculated at a daily temporal resolution for each variable using harmonic analysis with a three-harmonic fit to the observations. The stitched together, hourly NH-10 time series and seasonal cycles are available via Zenodo at https://doi.org/10.5281/zenodo.7582475.

**Specifications Table**

**Table:** 

Subject	Oceanography
Specific subject area	Hydrographic mooring data collected at station NH-10 off Newport, Oregon (44.64°N, 124.30°W)
Type of data	Hydrographic Figure
How the data were acquired	Primary temperature and practical salinity data were acquired using Sea-Bird Electronics (SBE) 16plus V2 SeaCAT (SBE-16) and 37 MicroCAT (SBE-37) CTD (Conductivity, Temperature, Depth) sensors and SBE-39 and SBE-56 sensors, Pacific Marine Environmental Laboratory Miniature Temperature Recorders (MTRs), and Vemco temperature loggers. Primary velocity data were acquired using Teledyne 300 kHz and 600 kHz Workhorse Sentinel Acoustic Doppler Current Profilers (ADCPs), 250 kHz and 500 kHz Sontek Acoustic Doppler Profilers (ADPs), and Nortek Aquadopp point velocity meters.
Data format	Raw Analyzed
Description of data collection	The secondary data set consists of NetCDF files that contain analyzed hydrographic mooring data collected at NH-10 from 1997 - 2021; derived best-fit seasonal cycles, calculated using harmonic analysis; and associated linear regression coefficients. It also includes example MATLAB® and R scripts that show how to read the data files, plot time series, and calculate seasonal cycles using the provided regression coefficients.
Data source location	Institution: Oregon State University City/Town/Region: Corvallis, Oregon Country: USA Mooring location: 44.64°N, 124.30°W Primary data sources: Biological and Chemical Oceanography Data Management Office (https://www.bco-dmo.org/dataset/2458; https://www.bco-dmo.org/dataset/2459). The National Data Buoy Center (https://www.ndbc.noaa.gov/station_page.php?station=46094). The Ocean Observatories Initiative (https://thredds.dataexplorer.oceanobservatories.org/thredds/catalog/ooigoldcopy/public/catalog.html)
Data accessibility	The primary and secondary data described here are available at: Repository name: Zenodo Data identification number: 10.5281/zenodo.7582475 Direct dataset link: https://doi.org/10.5281/zenodo.7582475

## Value of the Data

• With more than two decades of hourly observations, the data presented here can, for example, be used to better understand local and basin-scale forcing of physical [1,2] and biological [3] coastal upwelling processes at intra-seasonal, seasonal, interannual, and decadal time scales.
• Long time series such as those presented here are necessary for the detection of long-term changes and trends embedded within diurnal, seasonal and interannual variability.
• For each variable at each depth, the long time series can be used to create a best-fit seasonal cycle that can be subtracted from individual measurements to form anomaly time series that, in turn, can be compared with other oceanographic and marine ecosystem measurements.
• These time series can be used to validate and verify numerical operational and climate models [4,5] as well as derived data products [6].

## 1 Objective

Much of the temperature, practical salinity, and velocity data collected by the NOPP, OrCOOS and NANOOS NH-10 moorings were previously not publicly available. This article not only makes the data publicly available for the first time but stitches the time series together into data sets that span more than two decades. An additional objective is to provide value-added seasonal cycles calculated for each variable. Taken together, data collected by these research programs have the potential to contribute synergistically to a better understanding of biological and physical processes associated with coastal upwelling systems at a variety of time scales.

## 2 Data Description

**Fig. 1:**
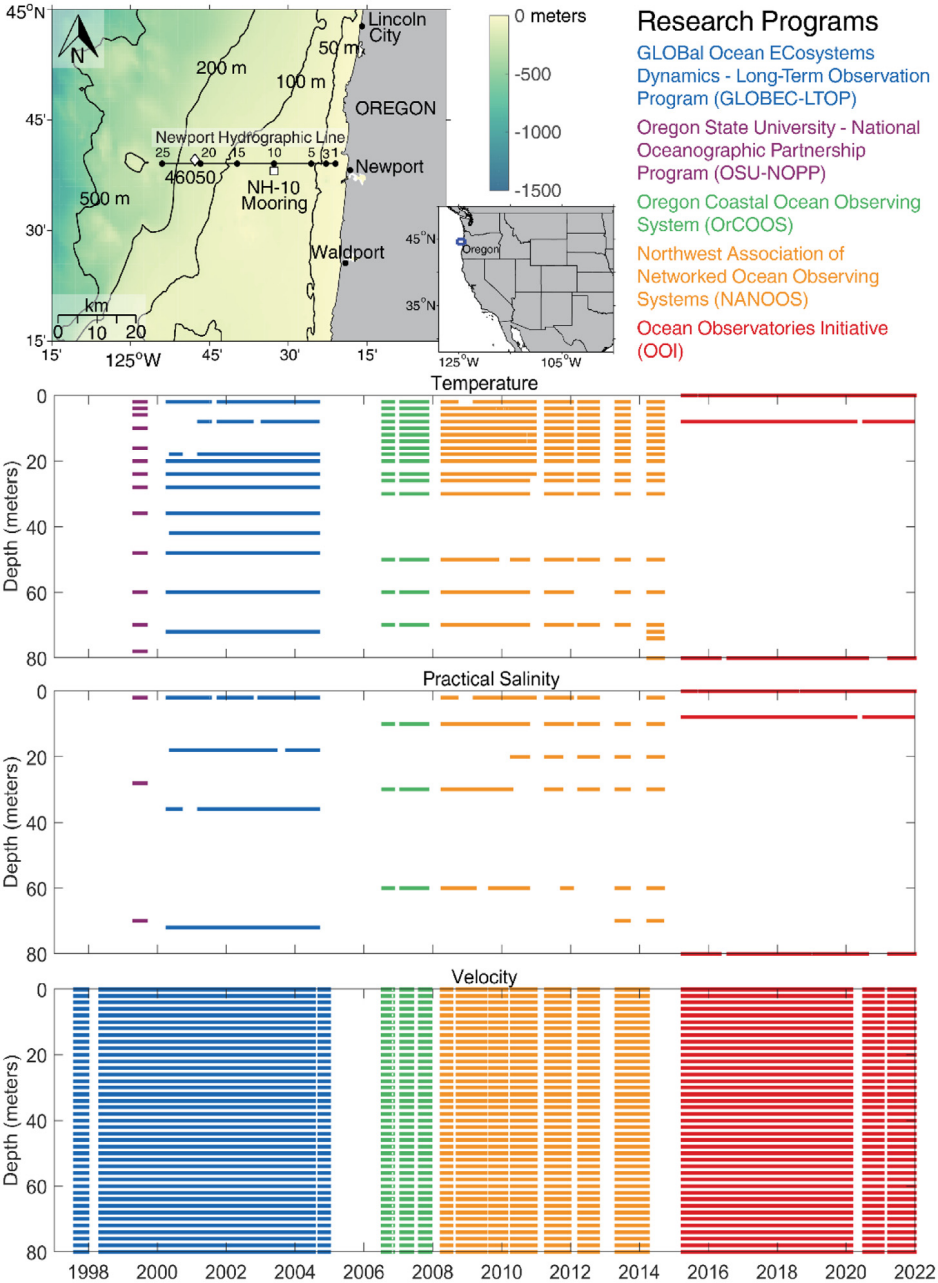
Top left: a regional map showing GEBCO bathymetry [8] and the NH-10 mooring location (white square), 18.5 km west of Newport, OR and slightly south of the Newport Hydrographic Line (NHL). Labeled NHL stations (1, 3, 5, 10, 15, 20, 25) correspond to the station distance from shore in nautical miles. The 50, 100, 200, and 500 meter isobaths are shown as well as National Data Buoy Center buoy 46050 (white diamond). The inset map shows the Western United States with the geographic bounds of the regional map indicated as a blue box off the Oregon coast. The lower three panels show data availability in time and depth for the temperature, practical salinity, and velocity time series at NH-10, respectively. Color indicates the research program responsible for collecting the mooring data. NH-10 was first deployed with velocity only in 1997, funded by GLOBEC. Temperature and salinity measurements were started jointly by GLOBEC and NOPP in April 1999, then continued with GLOBEC, OrCOOS, NANOOS, and OOI funding as shown.

**Fig. 2:**
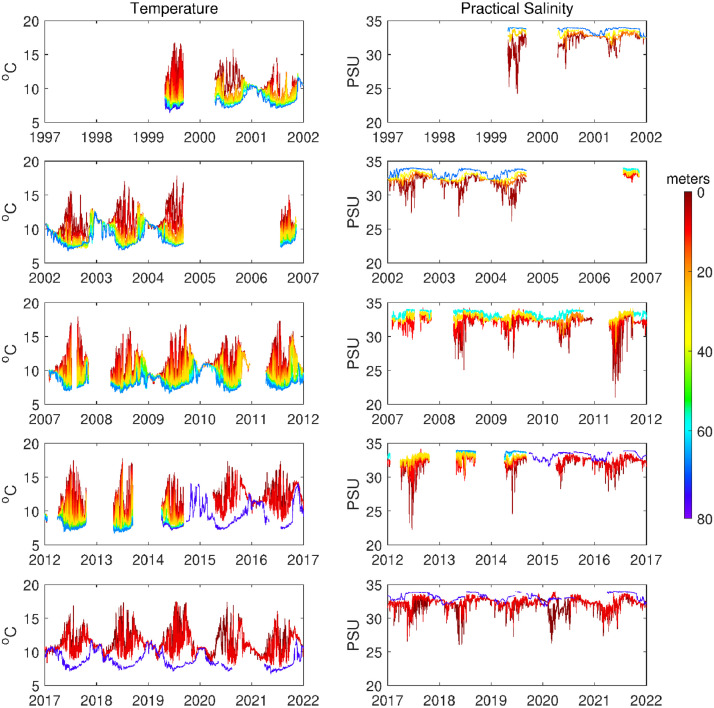
Hourly time series at NH-10 for temperature (left panels) and practical salinity (right panels) for all available depths from April 1999 – December 2021. Warmer colors indicate shallower depths. Cooler colors indicate deeper depths. Most gaps in the time series are the result of moorings breaking loose from their anchors during intense winter storms. Other gaps can be attributed to failed sensors or the funding gap that occurred between the end of the GLOBEC program and the start of OrCOOS.

**Fig. 3:**
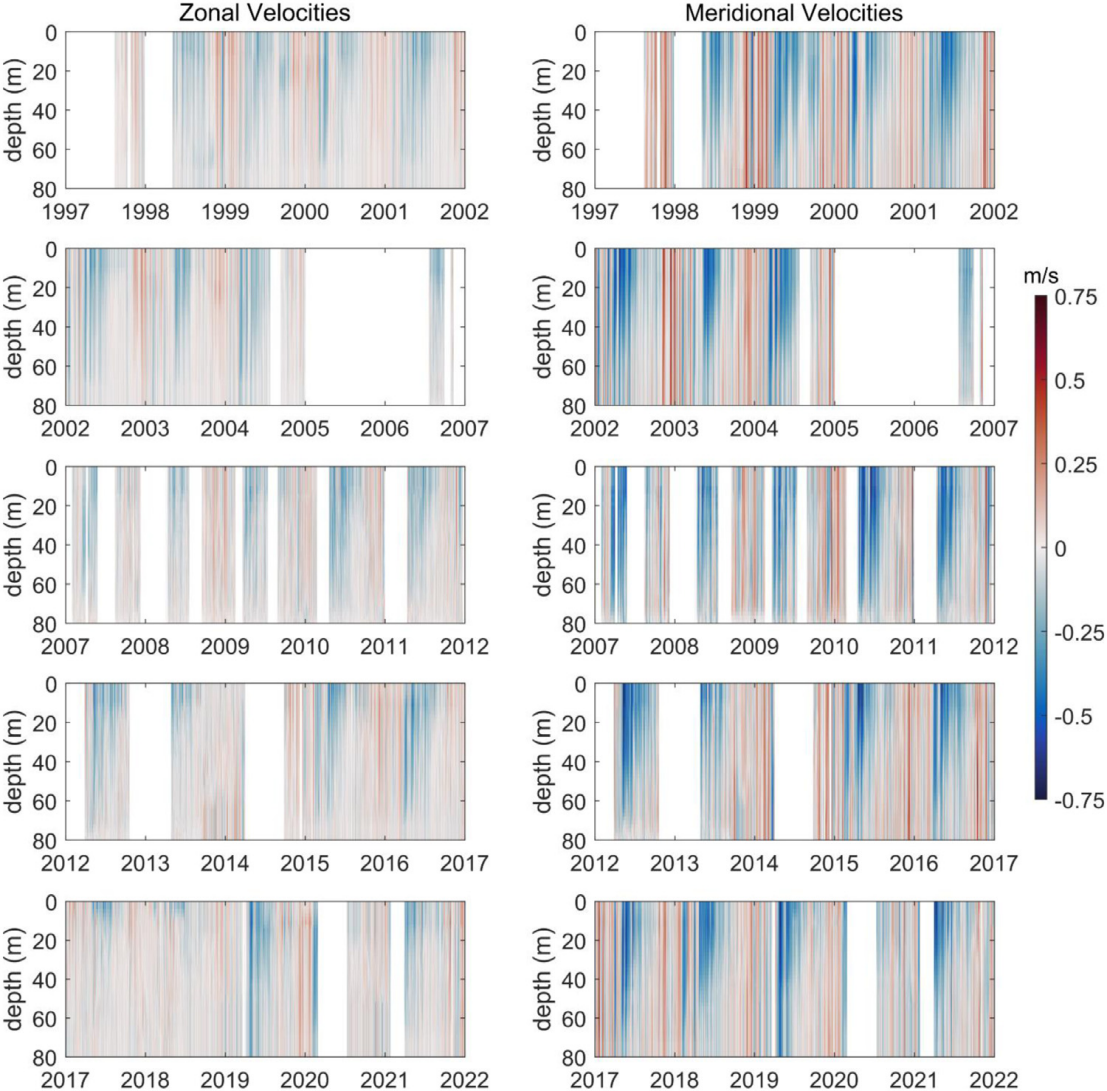
Time series of zonal (left panels) and meridional (right panels) velocity at NH-10 for all depths from August 1997 – December 2021. Most gaps in the time series are the result of moorings breaking loose from their anchors during intense winter storms or failed sensors. The 2005 to mid-2006 data gap is due to a lapse in funding that occurred between the end of the GLOBEC program and the start of OrCOOS.

**Fig. 4:**
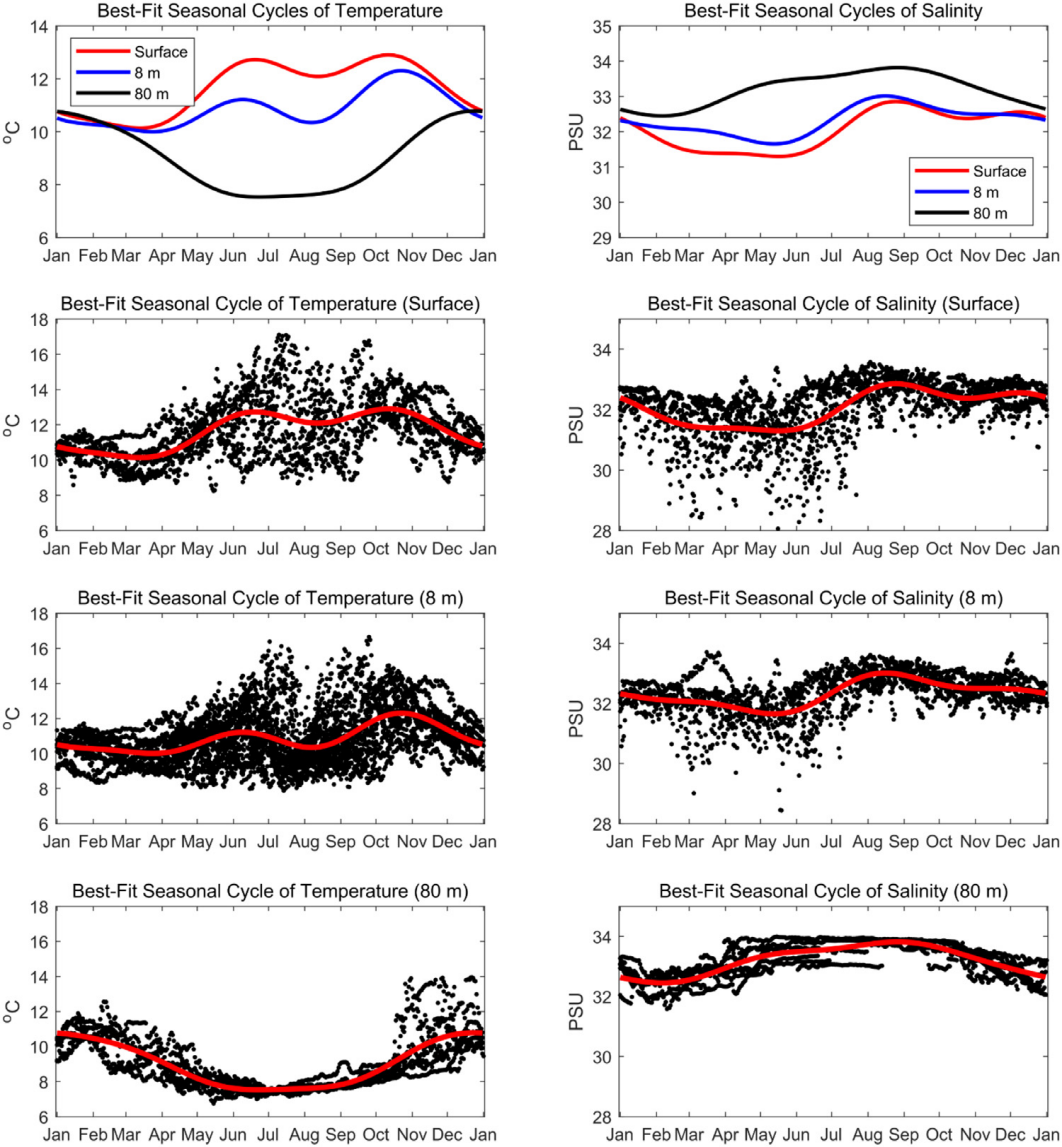
Top row: best-fit seasonal cycles for NH-10 temperature (left) and practical salinity (right) at the surface and at 8 and 80 meters depth (red, blue, and black, respectively). Lower three rows: best-fit seasonal cycles of temperature (left) and practical salinity (right) in red and daily averaged data from which the seasonal cycles are derived at each depth shown as black dots. Note the vertical scale differs in top row versus lower rows. The best-fit seasonal cycles are based on a three-harmonic fit (0, 1, 2, and 3 cycles per year) to the daily averaged time series from April 1999 – December 2021.

**Fig. 5:**
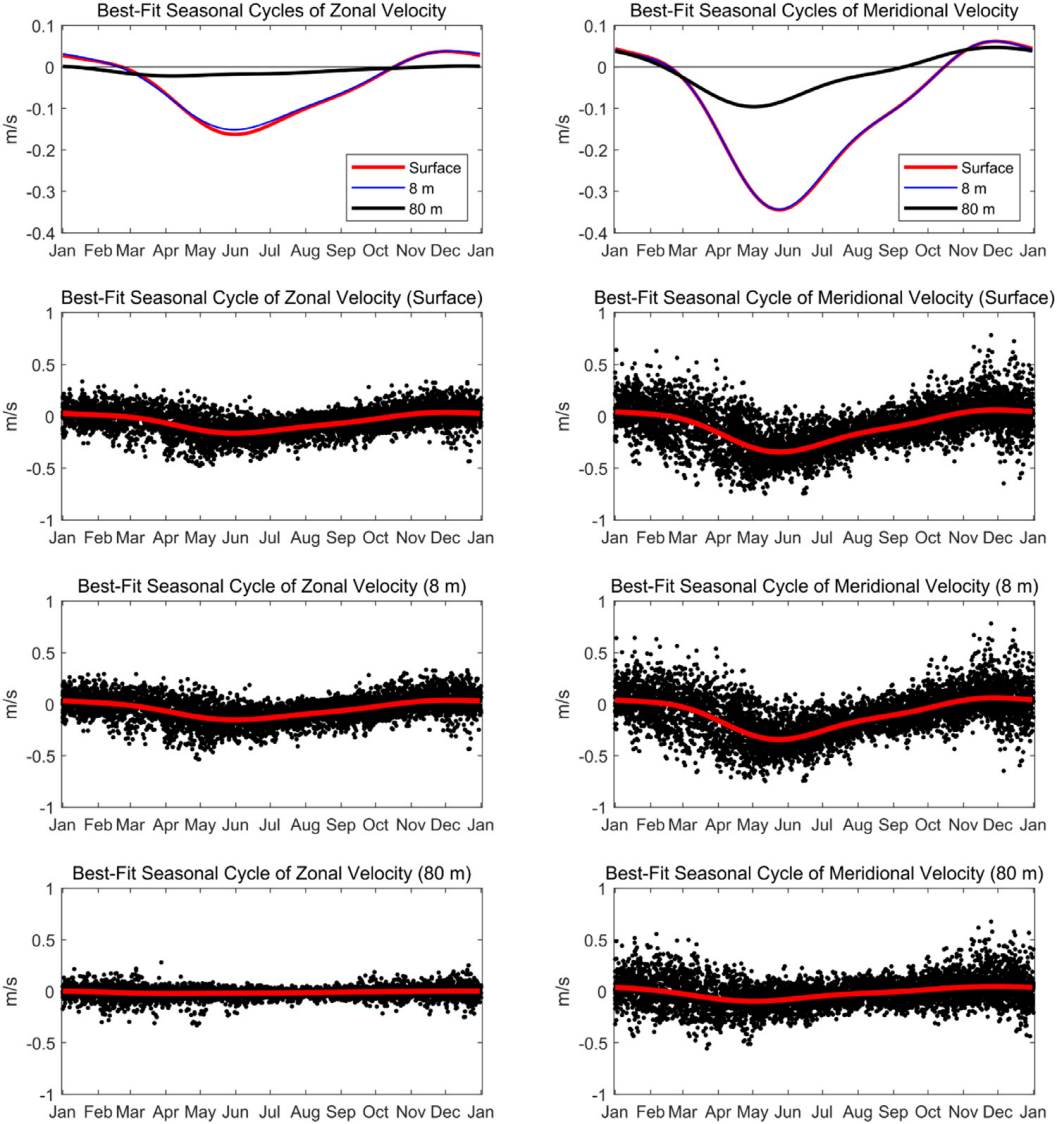
Top row: best-fit seasonal cycles for NH-10 zonal (left) and meridional (right) velocities at the surface and at 8 and 80 meters depth (red, blue, and black, respectively). Lower three rows: best-fit seasonal cycles of zonal (left) and meridional (right) velocities in red and daily averaged data from which the seasonal cycles are derived at each depth shown as black dots. Note the vertical scale differs in top row versus lower rows. The best-fit seasonal cycles are based on a three-harmonic fit (0, 1, 2, and 3 cycles per year) to the daily averaged time series from August 1997 – December 2021.

**Fig. 6:**
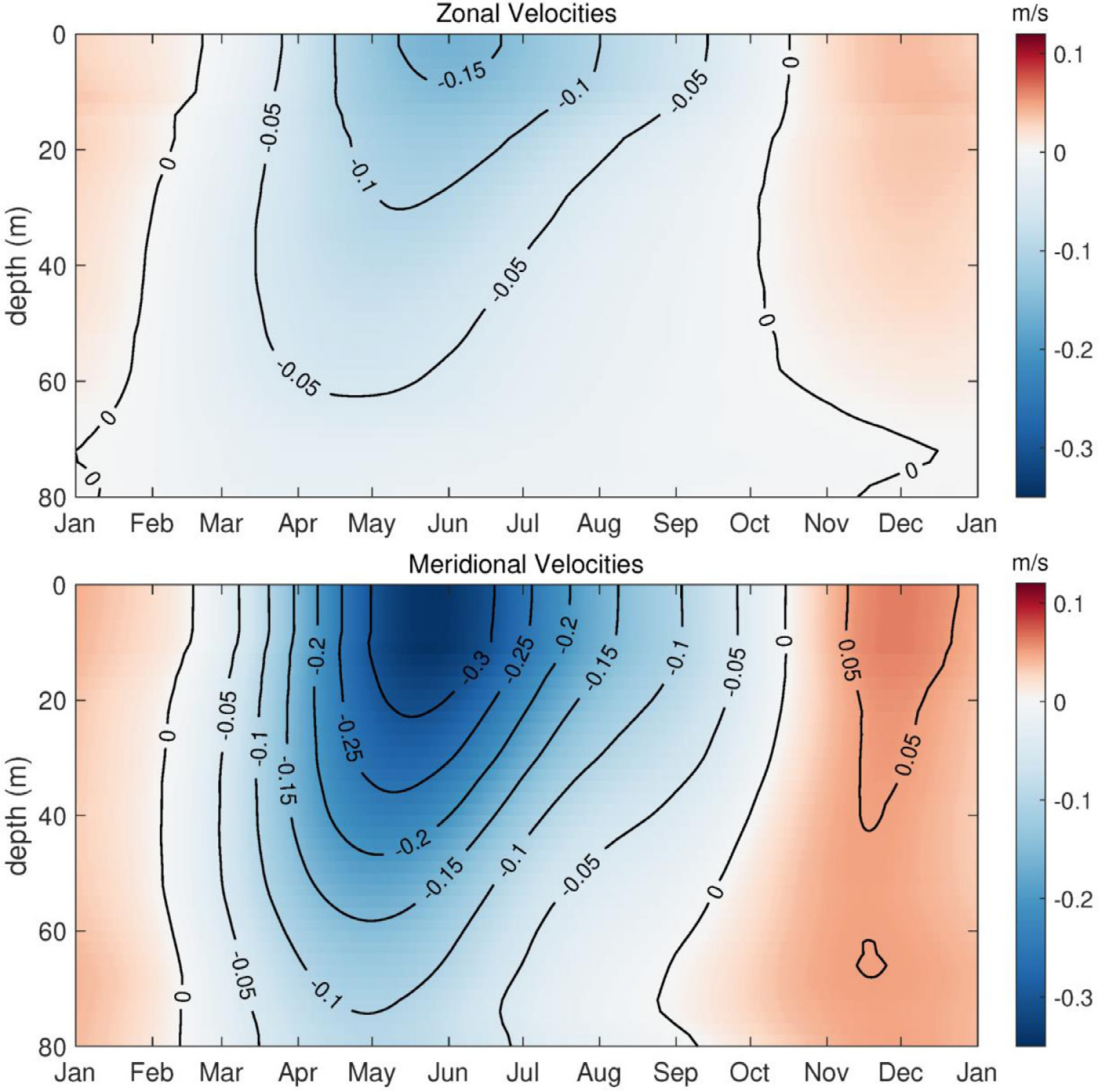
Best-fit seasonal cycles for NH-10 full water column zonal and meridional velocities (top and bottom panels, respectively). The best-fit seasonal cycles are based on a three-harmonic fit (0, 1, 2, and 3 cycles per year) to the daily averaged time series from August 1997 – December 2021.

The NH-10 station is located at approximately 44.64°N, 124.30°W, 10 nautical miles or 18.5 km west of Newport, Oregon on the 81-meter isobath (Fig. 1). The full water column temperature, practical salinity, and velocity mooring data collected at NH-10 and derived climatologies described here are available via Zenodo at https://doi.org/10.5281/zenodo.7582475 [7]. The data set includes three NetCDF files that follow CF (Climate and Forecast) metadata conventions. *nh10_hourly_data_1997_2021.nc* contains hourly temperature, practical salinity, and velocity data at 41 depths from 0 to 80 meters between August 1997 and December 2021 (Figs. 1, 2, and 3). *nh10_climatologies.nc* contains climatologies, calculated at a daily temporal resolution using harmonic analysis over the 25-year period 1997 to 2021 for meridional and zonal velocities, and the 23-year period 1999 to 2021 for temperature and practical salinity. Figs. 4 and 5 show temperature and practical salinity, and meridional and zonal velocity climatologies, respectively, for data at three discrete depths as well as the daily mean data (black dots) used to calculate the climatologies. Fig. 6 shows the meridional and zonal velocity climatologies throughout the water column. *nh10_climatology_coefficients.nc* contains the associated three-harmonic linear regression model coefficients for all four variables. Using the regression coefficients, investigators can create seasonal cycles at a temporal resolution that suits their particular needs.

## 3 Experimental Design, Materials and Methods

### 3.1 GLOBEC-LTOP

**Fig. 7:**
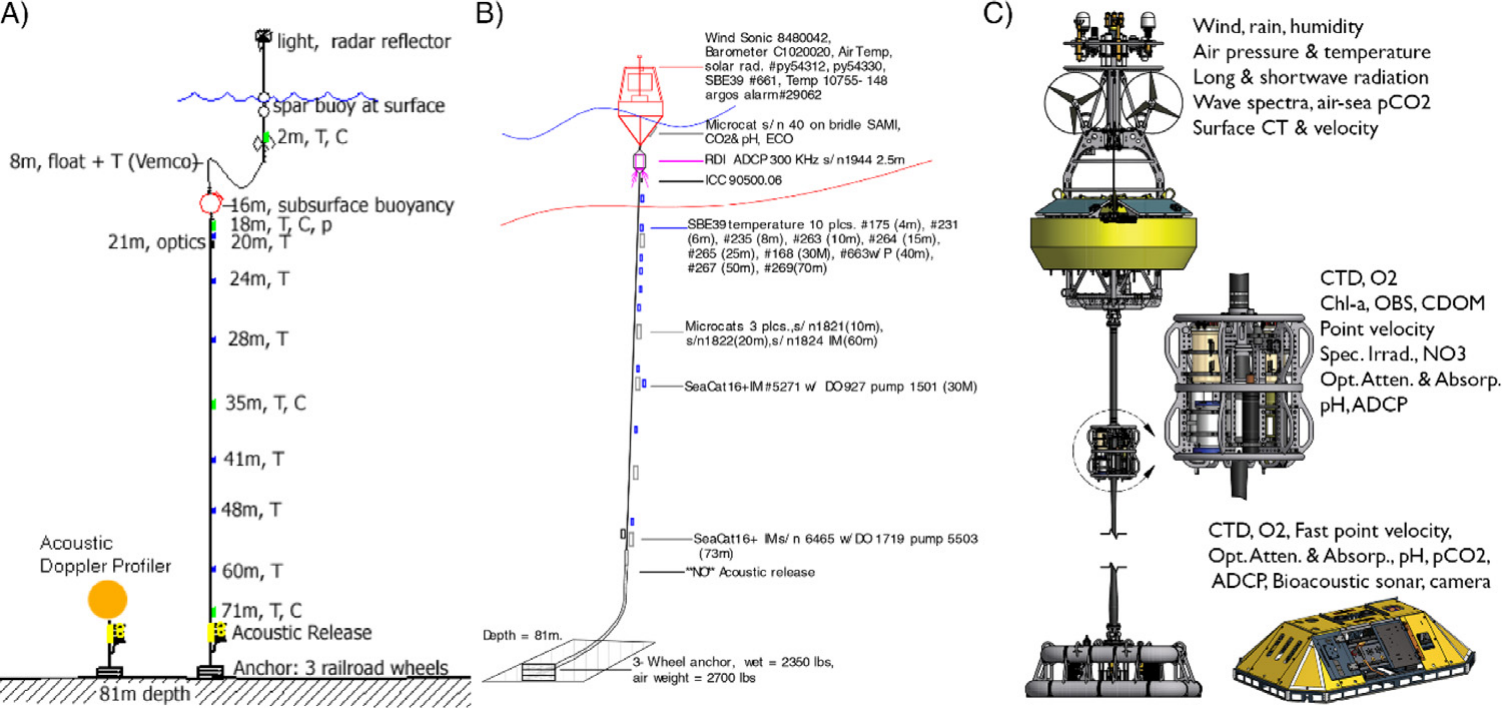
NH-10 mooring diagrams. (A) The GLOBEC (2000-2004) NH-10 mooring and Acoustic Doppler Profiler. (B) An example of a NANOOS (2008-2014) NH-10 mooring that was deployed in April 2010. C) The OOI Oregon Shelf mooring (2014-present) located at NH-10 and the co-located seafloor platform known as the Oregon Shelf Benthic Experiment Package. Diagrams shown in Panel C adapted with permission from diagrams available at https://oceanobservatories.org.

The National Oceanic and Atmospheric Administration (NOAA) and National Science Foundation (NSF) funded the Global Ocean Ecosystems Dynamics–Long Term Observational Program (GLOBEC-LTOP) [9], which investigated the effects of climate variability and climate change on the distribution and abundance of marine organisms in coastal ecosystems. GLOBEC-LTOP maintained a mooring at the NH-10 location, slightly south of the Newport Hydrographic Line (NHL; 44.652°N, 124.1 – 124.65°W) at approximately 44.64°N, 124.30°W, on the 81-meter isobath. A subsurface mooring was first deployed at NH-10 in August 1997 (Figs. 1 and 7). This mooring collected water column velocity data using an upward looking, Sontek Acoustic Doppler Profiler (ADP) from August 1997 to December 2004. Spring-summer deployments generally used 500 kHz systems with 2-meter bins, while winter deployments generally used 250 kHz systems with 4-meter bins. The ADP sampling strategy varied by deployment from 50 second averages every 150 seconds to 120 second averages every 300 seconds. Magnetic declination corrections were applied, and the data were assigned depths below the surface based on surface reflection and the deployment bin size. The data were hourly averaged and then low-pass filtered using a Lanczos filter [10] with a (40 hour)^−1^ half-power cut-off.

From April 2000 to September 2004 the GLOBEC-LTOP NH-10 surface mooring measured temperature at 11 depths (2, 8, 18, 20, 24, 28, 35, 41, 48, 60, and 71 meters below the water surface) using primarily Seabird sensors (SBE-16, SBE-37, SBE-39), except for the 8 meter depth sensors, which were Vemco sensors in a small “football” float on the tether between the subsurface float and the surface buoy. Seabird conductivity sensors (SBE-16, SBE-37) were deployed at 2, 18, 35, and 71 meters below the water surface (Fig. 7). All sensors had a 3-minute sample rate, except the Vemco, which had a 30-minute sample rate. The data were hourly averaged and low-pass filtered using a Lanczos filter with a (40 hour)^−1^ half-power cut-off.

### 3.2 OSU-NOPP

The aim of the Oregon State University (OSU) National Oceanographic Partnership Program (NOPP) was the development of nowcast and forecast numerical models for wind-driven coastal oceans. The project comprised modeling, data assimilation and observational activities along the Oregon coast. The observational program included coastal radar surface velocity measurements, ship-based hydrographic and velocity sampling off Newport, Oregon, and, from April to September 1999, moorings equipped with temperature and conductivity sensors throughout the water column deployed at NH-10 (Fig. 1). Pacific Marine Environmental Laboratory Miniature Temperature Recorders (MTRs) and SBE-16, SBE-37, and SBE-39 temperature sensors were deployed at 13 depths (2, 4, 6, 10, 16, 20, 24, 28, 36, 48, 60, 70, and 78 meters below the water surface). Conductivity was measured by SBE-16 and SBE-37 sensors located at 2, 28, and 70 meters below the water surface. All instruments had a 4-minute instantaneous sample rate.

### 3.3 OrCOOS and NANOOS

**Fig. 8:**
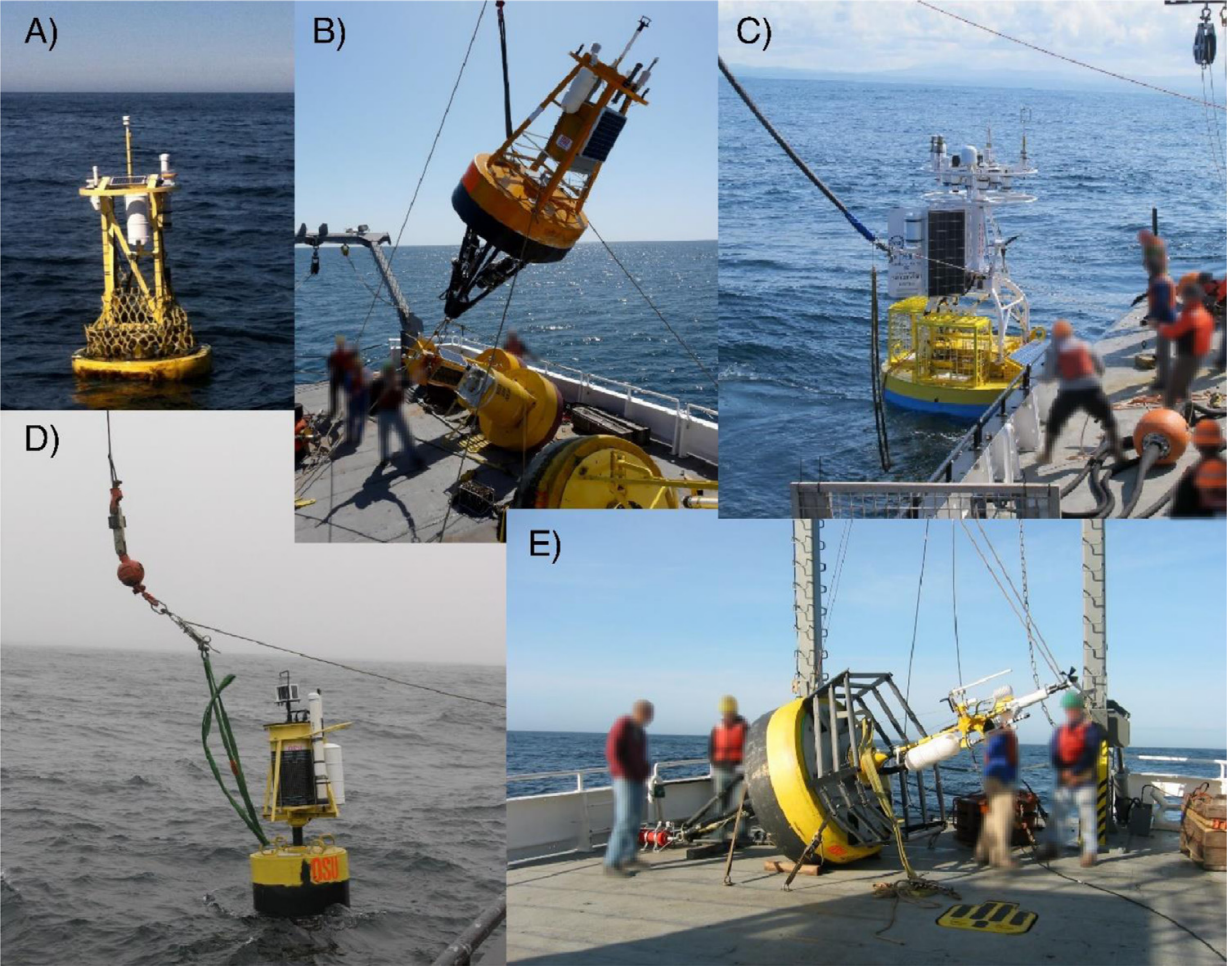
Photographs showing various versions of the NANOOS (panels A, B, D) and OrCOOS (panel E) NH-10 moorings. Examples of the NANOOS summer and winter moorings are shown in panels B and D, respectively. The presently deployed OOI Oregon Shelf mooring located at NH-10 is shown in panel C. Photographs provided by Oregon State University.

The overarching goal of the NOAA-funded Oregon Coastal Ocean Observing System (OrCOOS) was to establish and maintain a long-term ocean observing system along the Oregon coast that included near real-time ocean observing and modeling systems. Examples of such systems included moorings, ocean gliders, a land-based surface current mapping and wave detection radar array, and “nowcast” and “forecast” fields of sea surface currents and temperature derived from a coupled ROMS (Regional Ocean Modeling System) model. To help achieve this goal, OrCOOS re-deployed the surface mooring at NH-10 in July 2006 (Figs. 1, 7, and 8). The mooring included SBE-37 and SBE-39 temperature sensors deployed at 2, 4, 6, 8, 10, 12, 16, 20, 26, 30, 40, 50, 60, and 70 meters below the water surface and SBE-37 conductivity sensors at 10, 30, and 60 meters below the water surface. A downward looking Teledyne RD Instruments 300 kHz Workhorse Sentinel Acoustic Doppler Current Profiler (ADCP) was deployed beneath the mooring bridle at about 2.5 meters depth. ADCPs deployed between July 2006 and Dec 2007 were configured to record ensemble averages at either 2- or 4-minute intervals with ping rates from 8 to 27 seconds. All deployments had a 2-meter bin size.

The OrCOOS infrastructure became part of the NOAA funded Northwest Association of Networked Ocean Observing Systems (NANOOS), the U.S. Integrated Ocean Observing System Regional Association in the Pacific Northwest, in 2008, after which NANOOS operated and maintained the NH-10 mooring. NH-10 moorings were recovered and deployed approximately every six months between 2008 and 2014 (Fig. 1) with the NSF-funded Center for Coastal Margin Observation & Prediction (CMOP) providing ship time support. While the summer and winter NANOOS NH-10 mooring designs differed (Fig. 8), efforts were made to keep data collection consistent across all deployments. Moorings typically included 14 SBE-16, SBE-37, and SBE-39 temperature sensors deployed at 2, 4, 6, 8, 10, 12, 16, 20, 26, 30, 40, 50, 60, and 70 meters below the water surface. SBE-16 and SBE-37 conductivity sensors were typically deployed at 2, 10, 20, 30, and 60 meters below the water surface. All instruments had a 4-minute instantaneous sample rate. A downward looking Teledyne RD Instruments 300 kHz Workhorse Sentinel ADCP was deployed beneath the mooring bridle at about 2.5 meters depth (Fig. 7). ADCPs deployed between July 2008 and September 2014 were configured to have 2-meter bin sizes and to record 4-minute ensemble averages with a 15-second ping rate. Magnetic declination corrections were applied to all ADCP data collected between July 2006 and September 2014.

### 3.4 OOI

The NSF-funded Ocean Observatories Initiative (OOI) Endurance Array comprises instrumented buoys, profilers, ocean gliders, and seafloor platforms deployed off the coasts of Oregon and Washington [11]. These platforms collect physical, chemical, and biological data from the seafloor to the air-sea interface with the goal of better understanding processes impacting coastal upwelling, climate variability, ecosystem dynamics, and fisheries. The OOI first deployed a seafloor platform known as the Oregon Shelf Benthic Experiment Package (BEP) and the Oregon Shelf mooring at the NH-10 site in September 2014 and April 2015, respectively (Figs. 1, 7, and 8). The BEP includes an upward looking Teledyne RD Instruments 300 kHz Workhorse Sentinel ADCP and a SBE-16 CTD. Both instruments are deployed at a depth of 80 meters and have a 1 Hz sample rate. The BEP ADCP has a 4-meter bin size. The surface mooring includes a SBE-37 conductivity and temperature sensor deployed at 1.1 meters below the water surface that has a 1-minute instantaneous sample rate and a Nortek Aquadopp point velocity meter, deployed at 1 meter depth, that records an ensemble average (sampling at 1 Hz for 3 minutes) every 15 minutes. A downward looking Teledyne RD Instruments 600 kHz Workhorse Sentinel ADCP and a SBE-16 CTD are deployed at 7 meters depth on the near-surface instrument frame (Fig. 7). The SBE-16 records an ensemble average (sampling every 10 seconds for 3 minutes) every 15 minutes. The downward looking surface mooring ADCP records an ensemble average (pinging at 1 Hz for 3 minutes) every 15 minutes and has a 1-meter bin size. Magnetic declination corrections were applied to all OOI ADCP and Nortek Aquadopp velocity data collected between September 2014 and December 2021. The details of how the OOI applies magnetic declination corrections are available online at https://github.com/oceanobservatories/ion-functions/blob/master/ion_functions/data/adcp_functions.py. Information on OOI sampling strategies is available at https://oceanobservatories.org/knowledgebase/how-does-the-observatory-control-instrument-sampling/.

### 3.5 Data processing

For the purpose of this article, all velocity data were hourly averaged, except for the ADCP data collected between August 1997 and December 2004. The only publicly available data from the GLOBEC ADCP time series collected between August 1997 and December 2004 have already been low-pass filtered using a Lanczos filter with a (40 hour)^−1^ half-power cut-off and then decimated to a 6-hour resolution. For this article, those low-pass filtered GLOBEC data were linearly interpolated in time and space to create hourly time series at 2-meter reference depths between 0 and 80 meters.

All ADCP data were then linearly extrapolated from the shallowest reference depth to the surface and the deepest reference depth to the seafloor and linearly interpolated to reference depths at 2-meter intervals from 0 to 80 meters depth. For the OOI period (September 2014 to December 2021), data collected by the upward looking BEP ADCP were included in the time series when available. During periods when the BEP data were not available, data from the downward looking ADCP, deployed at 7 meters depth on the surface mooring, were included in the time series. During the OOI period, velocity data were linearly interpolated between the Nortek Aquadopp time series located at 1 meter depth and either the shallowest upward looking BEP ADCP bin depth or the first downward looking ADCP bin depth.

The majority of the temperature and practical salinity data were hourly averaged and mapped to the closest 2-meter depth between 0 and 80 meters depth. The exception was GLOBEC data collected between April 2000 and September 2004, which had already been low-pass filtered using a Lanczos filter with a (40 -hour)^−1^ half-power cut-off and then decimated to a 6-hour resolution similarly to the GLOBEC ADCP data described above. These low-pass filtered GLOBEC data were linearly interpolated in time to create hourly time series and then mapped to the closest 2-meter depth between 0 and 80 meters.

### 3.6 Best-fit seasonal cycles

To estimate the best-fit seasonal cycle for each variable at each depth, first, daily averaged temperature, practical salinity and meridional and zonal velocity time series were calculated from the hourly averaged time series. Then a seven-parameter linear regression model consisting of a constant plus three harmonics (0, 1, 2, and 3 cycles per year) was fitted to each multiyear, daily averaged time series using linear regression. The regression model for the best-fit seasonal cycle ${\hat{y}}_{seasonal}\left(t\right)$ for a particular variable (e.g., temperature at one depth), can be written as follows:

$$\begin{array}{ccc} {\hat{y}}_{seasonal}\left(t\right) & = & \;{\hat{\beta }}_{0}+{\hat{\beta }}_{1}\text{sin}\left(\omega t\right)\;+{\hat{\beta }}_{2}\text{cos}\left(\omega t\right)\;+{\hat{\beta }}_{3}\text{sin}\left(2\omega t\right)\;+{\hat{\beta }}_{4}\text{cos}\left(2\omega t\right)\\ & & +\;{\hat{\beta }}_{5}\text{sin}\left(3\omega t\right)\;+{\hat{\beta }}_{6}\text{cos}\left(3\omega t\right) \end{array}$$

where *t* is time, the ${\hat{\beta }}_{m}$ are the best-fit parameters determined by linear regression of the above model to the observed time series *y*(*t*), and ω = 2π/(365.2422 days), which is the angular frequency of rotation corresponding to a period of one year on Earth's surface. The decision to include three harmonics was based on extra sum of squares tests, which indicated higher harmonics were not statistically significant.

## Ethics Statements

The data sets described here involved no human subjects, animal experiments or social media platforms.

## CRediT authorship contribution statement

**Craig M. Risien:** Writing – original draft, Data curation, Validation. **Brandy T. Cervantes:** Writing – review & editing, Data curation, Validation. **Melanie R. Fewings:** Supervision, Writing – review & editing. **John A. Barth:** Supervision, Writing – review & editing, Data curation, Validation. **P. Michael Kosro:** Supervision, Writing – review & editing, Data curation, Validation.

## Declaration of Competing Interest

The authors declare that they have no known competing financial interests or personal relationships that could have appeared to influence the work reported in this paper.

## Data Availability

A Stitch in Time: Combining More than Two Decades of Mooring Data from the Central Oregon Shelf (Original data) (Zenodo).
